# Effects of wound dressing based on the combination of silver@curcumin nanoparticles and electrospun chitosan nanofibers on wound healing

**DOI:** 10.1080/21655979.2022.2031415

**Published:** 2022-02-09

**Authors:** Chuanliang Liu, Yuhua Zhu, Xuejie Lun, Huan Sheng, Anquan Yan

**Affiliations:** a The First Department of Health Care, Weifang People’s Hospital, Weifang, Shandong Province, China; b Department of Child Health Care, Anqiu Women and Children’s Hospital, Shandong Province, China; c Department of Operation Room, Weifang Municipal Hospital, Weifang, Shandong Province, China; d College of Pharmacy, Weifang Medical University, Weifang, Shandong Province, China

**Keywords:** Wound dressing, electrospun nanofibrous, silver nanoparticles, curcumin, antibacteria

## Abstract

Healing of various skin wounds is a lengthy process and often combined with bacterial infection and scar formation. Biomimetic electrospun nanofibrous wound dressing loaded with materials that possess properties of dual antibacterial and tissue repair would be developed to address this problem. In this study, a composite chitosan electrospun nanofibrous material containing Cur@β-CD/AgNPs nanoparticles composed of silver and curcumin possessed synergic effects on antibacterial activity and wound healing. The developed functionalized silver nanoparticles showed effective activity against both Gram-negative and Gram-positive bacteria. In vivo, Cur@β-CD/AgNPs chitosan dressing displayed enhanced wound closure rates compared to commercial AquacelAg. Moreover, Cur@β-CD/AgNPs chitosan dressing contributed to the most uniform collagen distribution by Masson’s trichrome staining. In brief, Cur@β-CD/AgNPs chitosan nanofibers work as a potential wound dressing with antibacterial and antiscarring properties.

## Graphical Abstract

**Figure:**
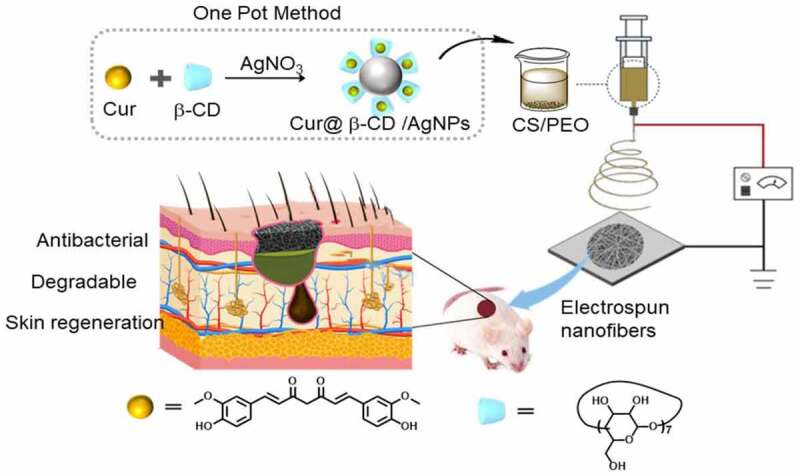

## 1. Introduction

As the incidence of cutaneous chronic wounds is on the rise, cutaneous chronic wounds are characterized by long lasting inflammation resulted from bacterial infection, diabetes mellitus, poor extracellular matrix deposition, and enhanced ability of proteases [1,2]. The rapid emergence of drug resistant microorganisms, which are increasing worldwide, poses a growing global healthcare threat to human health [3]. Disturbances to wound repair, often caused by the presence of stubborn bacterial infection, lead to major complications and delay tissue proliferation in the wound healing process [4,5].

As a result of the explosion of infectious diseases invited by deterioration of stubborn bacterial infection, efficient therapies with favorable safety and strong antibacterial activity are witnessing urgent demand [6]. Wound dressing treatment based on nanotechnology offers a remarkable chance to regulate the intricacy of chronic wound healing process via mimicking properties of extracellular matrix (ECM), fighting against antibiotic resistant bacteria and promoting ECM accumulation [7,8].

Due to favorable hemostatic activity, biocompatibility, biodegradability and antibacterial properties of natural polymer chitosan (CS), the electrospun polymeric nanofibers based on CS have been chosen as a wound dressing for pro-wound healing activities [9,10]. Silver nanoparticles (AgNPs), a kind of increasingly prevalent consumer product, are deemed to be one of the most prominent antibacterial agents via increasing membrane permeability of bacteria, denaturing bacterial proteins and interfering with DNA replication [11,12] AgNPs possessed broad-spectrum antibacterial ability, scarce bacterial resistance and low toxicity to mammal cells [2,13]. Curcumin (Cur) is a natural polyphenolic compound that isolated from rhizomes of Curcuma longa [14]. It possesses numerous health benefits such as anticancer, anti-oxidant, anti-inflammatory and antimicrobial properties with regard to less toxicity and low price [15].

In this work, in order to kill stubborn bacteria effectively and reduce silver toxicity, a nanometer electrospun nanofibrous wound dressing based on CS were developed which carried nanoparticles composed of silver in the core and curcumin on the surface. β-cyclodextrin (β-CD) increases the stability of nanomaterials and loads hydrophobic curcumin. Acid-responsive Cur@β-CD/AgNPs showd less toxicity and stronger antibacteria effects against *P. aeruginosa*, *S. aureus* and *E. coli* compared to commercial AgNPs dressing. Chitosan nanofiber could worked as a scaffold for the proliferation of fiberblast; both curcumin and degraded chitosan promote wound healing with less scar formation. In brief, the developed CS/Cur@β-CD/AgNPs wound dressing would be an upcoming method for intensifying antibacterial ability and promoting tissue repair in skin wound.

## 2. Materials and methods

### 2.1. Materials

Polyethylene oxide (PEO, molecular weight: 900 kD) and CS (molecular weight: 150 kD, ≥75% deacetylated) were purchased from Aladdin Co., Ltd (Shanghai, China). Silver nitrate (AgNO_3_, 99.9%), β-CD (99%) and Cur (98%) were purchased from J&K Chemical Company (Beijing, China). Sodium hydroxide (NaOH), acetic acid, dimethyl sulfoxide (DMSO) and other organic solvent were provided by Yantai Far East Fine Chemical (Shandong, China). LB broth and agar were obtained from Kamimi Yasuro Biological Technology Co., Ltd (Shanghai, China). 3-(4.5-dimethyl-thiazol-2-yl)-2.5-diphenyl tetrazolium bromide (MTT) was purchased from Lsolarbio (Beijing, China).

### 2.2. Synthesis of Cur@β-CD/AgNPs

All glassware were cleaned using freshly prepared aquilegia (3:1; HCl/HNO_3_) before used. In a typical experiment, 10 mM β-CD dissolved in 49.3 mL of water was added into a 100 mL flat-bottomed flask with a condenser [16]. Curcumin (20 mM, 250 μL) dissolved in DMSO was mixed with the above β-CD solution (30 mM) by vigorous stirring at 100°C. Then, 2.5 mL AgNO_3_ (10 mM) was added into the solution rapidly. Subsequently, 0.5 mL of K_2_CO_3_ (1.0 M) was added to the mixture lightly and adjusted pH to 10–12. The mixture was stirred for another 1 h at 100°C and cooled down to room temperature. Solution was centrifuged at 11,000 rpm for 15 min. After being washed with water, the Cur@β-CD/AgNPs were obtained by centrifugation at 11,000 rpm for 15 min, and the nanoparticles were dried by vacuum freeze for further use.

### 2.3. Fabrication of CS/PEO nanofiber

First, 3 wt% CS solution was prepared by dissolving CS (3 g) in 1% acetic acid. Subsequently, a certain quantity of Cur@β-CD/AgNPs and PEO (3 g) were added to the CS solution. Before electrospinning, a homogeneous solution containing 2 wt% Cur@β-CD/AgNPs (relative to the total weight of CS/PEO) was obtained by stirring overnight. The nanofibers were prepared using an electrospinning system (Ucalery, Yongkang Leye Technology Development Co. Ltd., Beijing, China). Typically, the prepared solution which was loaded into a 10 mL plastic syringe attached with 18-G stainless steel needle was fitted to the syringe pump of the electrospinning system with speed of 0.02 mm/min. High voltage was set up for 25 kV and the distance between collector device and needle was 25 cm. The rotational speed was 30 rpm. During the electrospinning process, the relative humidity and temperature were ranged from 25%-35% and 25–30°C, respectively [17].

### 2.4. Characterization of nanoparticles and nanofibers

Zeta potential and hydrodynamic diameter were gauged by Malvern Zeta/sizer Nano-ZSE (Zetasizer Nano ZS90; Malvern, UK). In addition, Cur@β-CD/AgNPs was placed in different solvents (PBS or DMEM) at room temperature to detect the size changes in different time periods. Transmission electron microscope (TEM, JEM-2010 HR, Japan) was performed to observe the morphology of Cur@β-CD/AgNPs. The morphologies of electrospun nanofibrous meshes were uncovered using scanning electron microscope (SEM, JSM701 F, JEOL, Japan). The incorporation of AgNPs be determined through atomic force microscope (AFM, Autoprobe CP Research, CA, USA). Fourier transform spectrophotometer (Thermo Scientific, USA) was employed to record the Fourier transform infrared (FTIR) spectra in the range of 4000–400 cm^−1^ using KBr pellets [18].

### 2.5. Assessment of swelling of nanofiber

The swelling proportion of electrospun nanofibrous meshes was performed by incubating nanofibrous membranes in PBS (pH 7.4, 37°C) for 24 h. Then, excess water cling to the surface of nanofibrous meshes was wiped off gently using filter paper and the nanofibers were weighed immediately. The swelling percentage was calculated according to previous report [19], where Ww represented the weight of wet nanofibers and the Wd represented the weight of dry nanofibers. All measurements were performed in triplicate.

### 2.6. In vitro degradation of the nanofibrous meshes

The in vitro degradation of electrospun nanofibrous meshes was measured using mass loss method. Briefly, a 6 mm diameter meshes were weighed (M0) and immersed in PBS (pH7.4) containing 1 mg/mL lysozyme for a period of 14 days. The lysozyme solution was changed every 2 days to maintain activity of lysozyme. The degraded meshes were dried completely, and their weights were recorded as Md. The degradation of nanofibrous meshes was calculated by the equation which had been reported previously [19].

### 2.7. Curcumin release study

In vitro curcumin cumulative release from Cur@β-CD/AgNPs nanoparticle was carried out by dialysis in PBS with different pH (pH 5.5 and 7.4) at 37°C. Briefly, 20 mg Cur@β-CD/AgNPs was suspended in PBS-containing Tween-80 (0.5% w/w) and ethanol (20%, w/w) and then the mixture was dialyzed using a dialysis bag (molecular weight cut off: 3500 Da, CelluSep, USA) in a shaking water bath at 200 rpm. At predetermined time points, 1 mL dialysis solution was withdrawn and 1 mL fresh buffer was added to maintain the constant volume. Curcumin concentration was measured with UV−visible spectrophotometer (UV-1700; Shimadzu, Kyoto, Japan) at 425 nm. All measurements were performed three times. The cumulative released amount of curcumin was calculated using the equation as previous report [20].

### 2.8. Hemocompatibility assay and cytotoxicity assay

2 mL mice blood in EP tube was centrifuged at 5000 rpm for 20 min and washed with PBS to collect red blood cells. Then, 100 μL red blood cells were incubated with 1 mL nanofiber (2 mg/mL) or distilled water (positive control) or PBS (negative control) at 37°C for 3 h, followed by centrifugation at 12,000 rpm for 15 min. OD value was measured at 540 nm, hemolysis rates were calculated according to previous report [21]. For cytotoxicity assay, L929 cells were inoculated into 96-well plates with 5 × 103 cells/well for 24 h, different concentrations of silver nanoparticles (50 μg/mL, 100 μg/mL, 150 μg/mL and 200 μg/mL) were added for 24 h, MTT solution (5 mg/mL) was added. After 4 h, cell culture medium was discarded and DMSO solution was added. The absorbance was measured at 490 nm (Multiskan Go, USA).

### 2.9. Antibacterial evaluation assay

The antibacterial assay of AquacelAg meshes and silver-loaded nanofibrous meshes was performed against *E. coli* (Gram-negative bacteria, number: 25,922), *S. aureus* (Gram-positive bacteria, number: 25,923) and *P. aeruginosa* (Gram-negative bacteria, number: 27,853). Fresh colonies were selected to prepare bacterial suspensions for each tested strain. The bacteria were shook at 250 rpm overnight at 37°C and the concentration of bacterial suspensions was adjusted to 1 × 108 CFU/mL. Then small pieces of nanofiber materials disinfected by ultraviolet light for 30 min were introduced to a tube with 3 mL (0.5% AgNPs) suspension of indicated bacteria, and CS/PEO was set as negative control. Each group was shook at 180 rpm at 37°C for 12 h, OD 600 values of solutions were monitored at 0, 2, 4, 6, 8, 10 and 12 h by Varioskan Flash (Multiskan Go; Thermo Fisher Scientific, MA, USA) [13]. For antibacterial ring test, the aforementioned three bacterial strains were cultured with sterilized LB broth at 37°C for 12 h, then the bacterial fluid was diluted with fresh LB to 1 × 10^8^ CFU/mL. The tested strains were evenly wiped onto culture plates, the sterile filter paper or nanofiber meshes with a diameter of 6 mm were placed in the bottom of culture plates which were incubated at 37°C for 12 h. Potential antibacterial activity was indicated by the diameter of inhibition zone.

### 2.10. In vivo wound healing assay

Wound healing assay was carried out using 25 g male Kunming mice (vitalriver, Beingjing, China). All animals were anesthetized with 3% pentobarbital sodium, then the back was shaved and a puncture appliance was used to create a 6 mm skin defects on the back of mice. To avoid the differences between individuals, all model mice were randomly divided into control, CS/PEO, AquacelAg, and Cur@β-CD/AgNPs meshes groups (n = 6). Nanofiber meshes of the same diameter were sterilized with ultraviolet light for 30 min, and each nanofiber meshes was placed at the wound site and secured with gauze. CS/PEO and AquacelAg was used as negative and positive control, respectively. Changes in wound area were measured at day 6 and 12, the wound area that remained exposed was measured for three times and the degree of wound healing was calculated according to a previous report [22].

### 2.11. Histological examination

Control and experimental mice were sacrificed at day 6 and 12 postsurgery, respectively. The tissue samples that comprised wound site and surrounding healthy tissue were removed and fixed in 4% paraformaldehyde, the samples of excised skin were embedded in paraffin and cut into 4 μm sections. Then, hematoxylin and eosin (H&E) staining as well as Masson’s trichrome (MT) staining were performed according to standard protocols. Histological analysis of H&E-stained or MT-stained sections at day 6 and 12 was assessed. The degree of necrosis, inflammatory infiltrates, hemorrhage, granulation tissue, epithelization, thickness of the epidermis and collagen deposition were evaluated [23].

### 2.12. Statistical analysis

Data were expressed as a mean ± standard deviation (SD). Student’s t test or analysis of variance (ANOVA) was used to comprise the means of each groups. Differences were considered significant if the P value was <0.05.

## 3. Results and discussion

To prevent intractable infection of skin wound and accelerate wound healing, we prepared electrospun CS/PEO nanofibrous meshes carrying nanoparticles which worked as an implantable delivery vehicle for the dual release of curcumin and silver. We found that a fast release of curcumin in 2 days. The antibacterial effects of the membranes against *P. aeruginosa*, *S. aureus* and *E. coli* displayed that the membranes caused an obvious inhibition zone upon loading with Cur@β-CD/AgNPs. In addition, the in vivo wound healing studies showed that CS/Cur@β-CD/AgNPs nanofibrous meshes led to accelerated wound healing with less scar formation as compared to commercial AquacelAg nanofibers as confirmed by MT stain.

### 3.1. Synthesis and characterization of Cur@β-CD/AgNPs

As previously noted, silver nanoparticles provided a potential opportunity for novel antibacterial therapies [24,25]. In this study, the multifunctional silver nanoparticle was prepared via one pot method involved in the use of curcumin, AgNO_3_ and β-CD. Herein, the -OH of curcumin was allowed to reduce Ag+ to Ag0 [26]. The β-CD worked as a shuttle for curcumin via a self-assembly mechanism [27]. The hydrophobic curcumin was loaded into the internal cavity of β-CD, while hydroxyl groups of β-CD improved curcumin solubility and stability. More importantly, the β-CD was also worked as a capping agent to stabilize AgNPs during the growth and nucleation process. It was well known that the capped ligands bearing hydroxyl groups stabilized the surface of AgNPs and prevented its aggregation [28].

**Figure 1.**
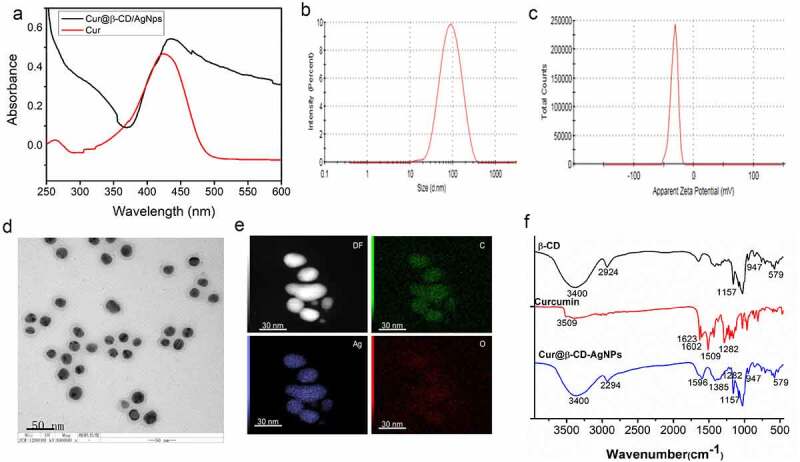
Characterization of Cur@β-CD/AgNPs. (a) Results of UV-vis absorption. (b) Hydrodynamic average diameter of Cur@β-CD/AgNPs by DSL. (c) Zeta potential of Cur@β-CD/AgNPs. (d) TEM images of Cur@β-CD/AgNPs. (e) Ag and C element mapping of Cur@β-CD/AgNP using EDX. (f) FTIR of β-CD, Curcumin and Cur@β-CD/AgNPs.

The color of the solution gradually changed from yellow to greenish yellow indicated successful synthesis of Cur@β-CD/AgNPs. In the results of UV-vis spectrum, a prominent absorption peak at around 439 nm could be observed, indicating a strong association of curcumin with AgNPs (Figure 1a). Results of dynamic light scattering (DLS) measurement showed that the multifunctional silver nanoparticles possessed a hydrodynamic diameter of 81.26 ± 5.24 nm with polydispersity index (PDI) of 0.19 (Figure 1b) and zeta potential of −31 mV (Figure 1c). Results of TEM revealed that multifunctional AgNPs showed a spherical shape with an average size of approximately 60 nm (Figure 1d). The sphericity of multifunctional silver nanoparticle during the formation of stable AgNPs was due to the hydroxyl groups in β-CD [28]. The elemental compositional analysis of the Cur@β-CD/AgNPs was performed using energy dispersive X-ray spectra (EDX). As displayed in Figure 1e, the silver and carbon elements are homogeneously distributed throughout the silver nanoparticles, which confirmed both the existence of Ag and organic capping agent (β-CD and Cur) within the nanoparticles. FTIR spectra was used to analyze the chemical composition of the silver nanoparticles, the results were shown in figure 1f. It was noted that the characteristic absorption peaks (-OH = 3400 cm^−1^, C-H = 2924 cm^−1^ and C-O-C = 1157 cm^−1^, 1–4 linkage = 947 cm^−1^, pyranose ring = 708 cm^−1^ and 579 cm^−1^) of β-CD were presented in Cur@β-CD/AgNPs [29]. The peak at 1602 cm^−1^, which corresponded to the C=C (benzene ring) stretching of curcumin, underwent a shift to 1596 cm^−1^ of Cur@β-CD/AgNPs, which was a fine evidence for the entrance of curcumin into β-CD cavities [27]. A new peak appeared at 1385 cm^−1^ might be due to inaction of Ag with the curcumin as reported earlier [30]. Results of FTIR analysis demonstrated that the prepared Cur@β-CD/AgNPs were capped with β-CD and curcumin successfully.

### 3.2. Cumulative curcumin released rapid from Cur@β-CD/AgNPs

**Figure 2.**
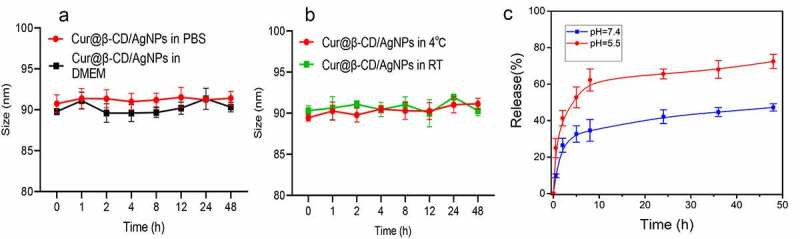
Results of cumulative curcumin release study. (a) Dynamic monitoring the size change of Cur@β-CD/AgNPs in PBS or DMEM (10% FBS) for 48 h. (b) Dynamic monitoring the size change of Cur@β-CD/AgNPs at 4°C or room temperature for 48 h. (c) Results of curcumin release from CUR@β-CD/AgNPs.

The stability of nanoparticles is an important parameter for biological applications and storage. Therefore, we investigated the stability of cur@β-CD/AgNPs. As shown in Figure 2a, the size of Cur@β-CD/AgNPs in PBS and DMEM medium (containing 10% FBS) fluctuated within the range of 90 nm. There was no significant change in the size of Cur@β-CD/AgNPs at 4°C and room temperature (Figure 2b). This shows excellent stability for biological applications. The pH of stage I of wound ulcers was approximately 5.5, which afforded for the treatment with wound dressings that released therapeutic components at acidic environment [31]. In vitro curcumin cumulative release from Cur@β-CD/AgNPs was carried out through dialysis in PBS with different pH values (5.5 and 7.4) at 37°C. The release amount of curcumin was plotted as cumulative release with time, very slow release rate of curcumin in pH 7.4 was observed (Figure 2c). In the initial burst release period, only about 47.2% curcumin was released from Cur@β-CD/AgNPs in 48 h. While the release rate of curcumin in the acidic environment (pH = 5.5) was significantly faster than that in physiological environment (pH = 7.4), approximately 52.8% drug was released at 5 h, approximately 72.4% of the drug was released in the whole period of 48 h (Figure 2). At alkalescent environment (pH 7.4), the silver ion cross-links contributed to the retention of curcumin in Cur@β-CD/AgNPs, whereas lower pH gave rise to the destruction of ionic interactions, protonation of curcumin, and resulting in release of curcumin.

### 3.3. Morphology of electrospun nanofibers

**Figure 3.**
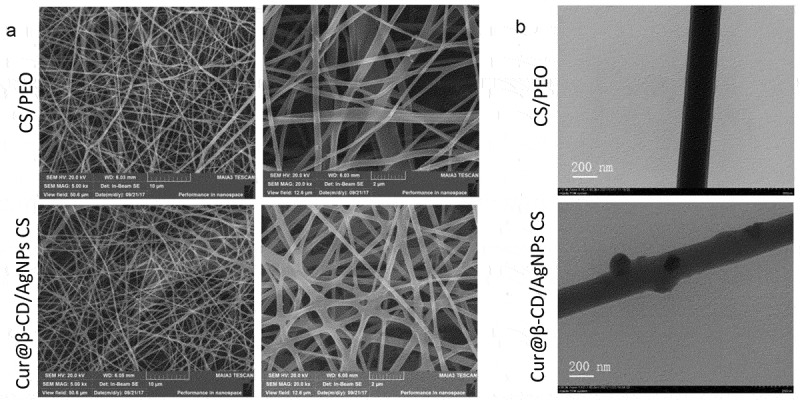
Characterization of Cur@β-CD/AgNPs meshes. (a) SEM images of CS/PEO meshes. (b) TEM images of Cur@β-CD/AgNPs in the CS/PEO nanofibers; scale bars represent 200 nm.

Pure CS could not form fibers and only beads or drops are deposited due to strong intramolecular and intermolecular hydrogen bonds between amino groups and hydroxyl [32]. With addition of PEO, the self-association of CS chains was disrupted by formation of additional hydrogen bond which improved chain entanglement and promoted the production of fibers [33]. The nanofibrous meshes were prepared by electrospun method as shown by SEM micrographs; uniform diameters (53.93 ± 17.07 nm) and smooth nanofibers were obtained when the ratio of CS to PEO was 1:1 (Figure 3a). With incorporation of 2% Cur@β-CD/AgNPs into the CS/PEO, the nanofibers still maintained smooth surface due to small size of Cur@β-CD/AgNPs nanoparticles, the diameter of electrospun nanofibers containing Cur@β-CD/AgNPs was 65.35 ± 23.14 nm (Figure 3a). In brief, the introduction of nanoparticles did not affect the appearance of the nanofibrous meshes. Results of TEM confirmed the successful incorporation of AgNPs (Figure 3b).

### 3.4. Assessment of swelling, biodegradation and biocompatibility

**Figure 4.**
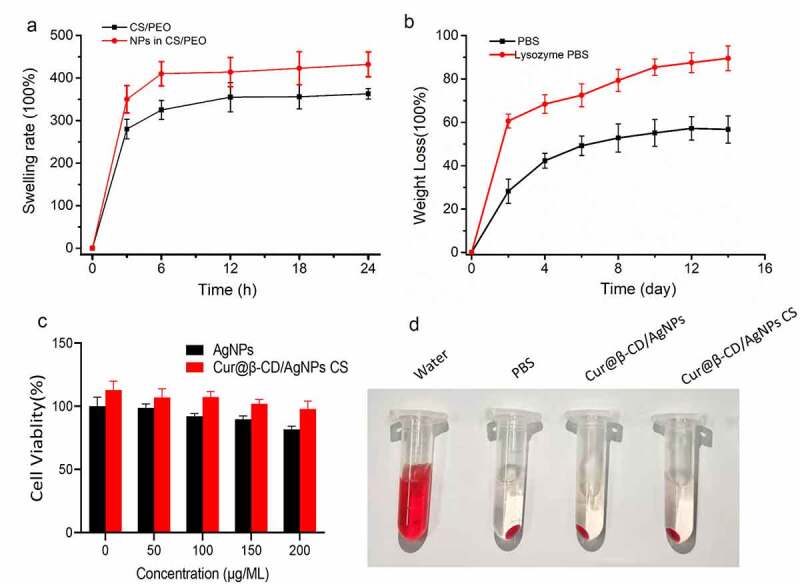
Swelling rate and degradation of Cur@β-CD/AgNPs. (a) The results of swelling test of Cur@β-CD/AgNPs. (b) Results of biodegradation abilities of Cur@β-CD/AgNPs nanofibers. (c) Viabilities of L929 cells at different concentration incubation with the nanofibers. (d) Hemolysis test of the nanofibers.

Biodegradation and swelling are important properties for the compatibility of biomaterial materials application [34]. Some researchers verified that swelling capacity of electrospun nanofibers was higher than that of cast films, which were conducive to cell growth, adhesion, and migration into internal space of three-dimensional porous scaffolds [35,36]. Swelling test was carried out to evaluate water absorption capacity of nanofibers. As presented in Figure 4a, the swelling ratio of CS/PEO nanofibers increased from 363% to 432% in the presence of 1% (m/m) Cur@β-CD/AgNPs, indicating that higher water absorption capability was acquired when Cur@β-CD/AgNPs nanoparticles were incorporated into electrospun nanofibers.

Wound dressing scaffolds should have ideal degradation rate to match new tissue regeneration. Regarding the fact that the degradation of CS was mainly degraded by lysozyme in human tissue, the degradations Cur@β-CD/AgNPs and CS/PEO nanofibers were tested by mass loss assay in physiological solution with or without lysozyme at 37°C for 14 days. As shown in Figure 4b, the Cur@β-CD/AgNPs nanoparticles loaded in the CS/PEO nanofibers were quite stable with 56.7% weight loss in PBS for 7 days. And the nanofibers were firm enough with 4 days of continuous soak in PBS. Cur@β-CD/AgNPs nanoparticles loaded in CS/PEO nanofibers exhibited a significant mass loss in presence of lysozyme due to degradation of CS by lysozyme (Figure 4b). These results suggested that the Cur@β-CD/AgNPs loaded in CS/PEO nanofibers were highly biodegradable as a wound dressing application.

Hemolysis tests are used to evaluate nanofiber’s blood compatibility, results are given as hemolysis rate values to the positive control. The results indicated that the hemolysis rate of 2 mg/mL nanofiber was lower than 3% compared with PBS (Figure 4c). According to the American Society for Testing and Materials (ASTM F 756–00, 2000), our results is considered as a safe hemostatic material. In addition, silver nanoparticles and nanofibrous showed negligible cytotoxicity. The survival rate of L929 cells co-incubated with silver nanoparticles and nanocomposites of different concentrations was greater than 80% after 24 hours, which showed no statistically significant difference from that of the positive control group (Figure 4d).

### 3.5. Excellent antibacterial activities of nano Cur@β-CD/AgNPs CS dressing

Optical density measurement was performed to evaluate antibacterial activities of Cur@β-CD/AgNPs against P. aeruginosa, E. coli and S. aureus. Aforementioned bacteria solutions were incubated with debris of nanomaterials at different concentrations and shook at 250 rpm overnight at 37°C.

**Figure 5.**
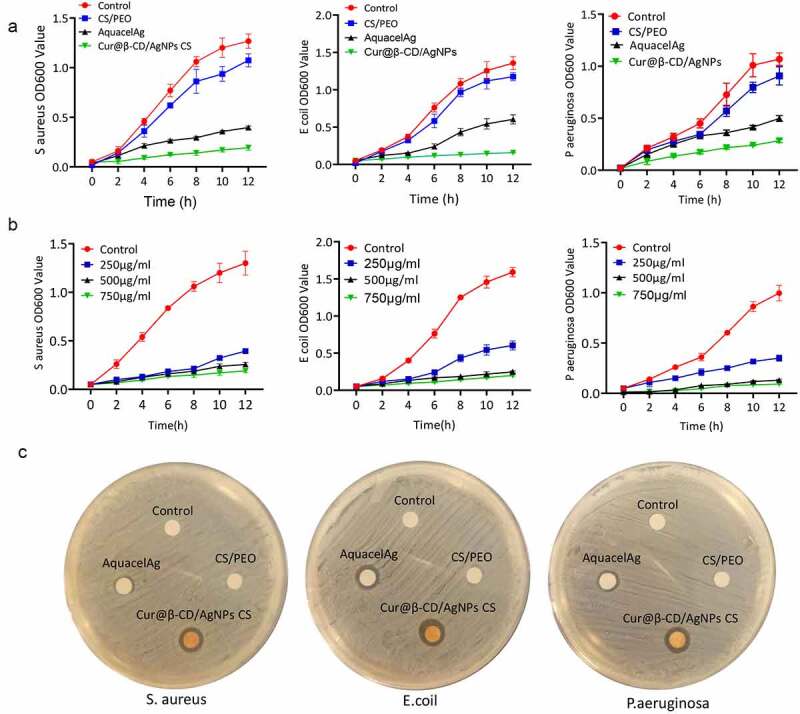
Antibacterial activity of CS/Cur@β-CD/AgNPs dressing. (a) Bacterial viability of *P. aeruginosa*, *S. aureus* and *E. coli* treated with four nanoparticles. (b) Bacterial viability of *P. aeruginosa*, *S. aureus* and *E. coli* treated with different concentrations of Cur@β-CD/AgNPs CS nanofibers. (c) The inhibition zone of nanofibers on *P. aeruginosa*, *S. aureus* and *E. coli*.

The absorbances at 600 nm after incubation were taken every 2 h during a period of 12 h. As shown in Figures 5A, 250 μg/mL Cur@β-CD/AgNPs nanofiber displayed stronger inhibitory effects on growth of three kinds of bacteria compared to AquacelAg. The viabilities of *P. aeruginosa*, *S. aureus* and *E. coli* were significantly decreased with the increasing concentration of Cur@β-CD/AgNPs CS nanofibers, indicating inhibitory effects were a dose-dependent manner (Figure 5b). What’s more, the content of Ag in Cur@β-CD/AgNPs was less than that of AquacelAg when both weights were equal, implying that combination of silver with curcumin possessed synergic effects on antibacterial activity. Due to polycationic nature, CS also showed antibacterial activity which resulted in leakage of proteinaceous and other intracellular constituents when it interacted with the negatively charged bacterial cell membrane. The antibacterial activities of nano Cur@β-CD/AgNPs CS dressing had also been confirmed by determining diameter of the zone of inhibition (Figure 5c). These data indicated that augmented antibacterial activities of nano Cur@β-CD/AgNPs CS dressing against *P. aeruginosa*, *S. aureus* and *E. coli* were achieved.

### 3.6. Evaluation of the wound healing ability in vivo

**Figure 6.**
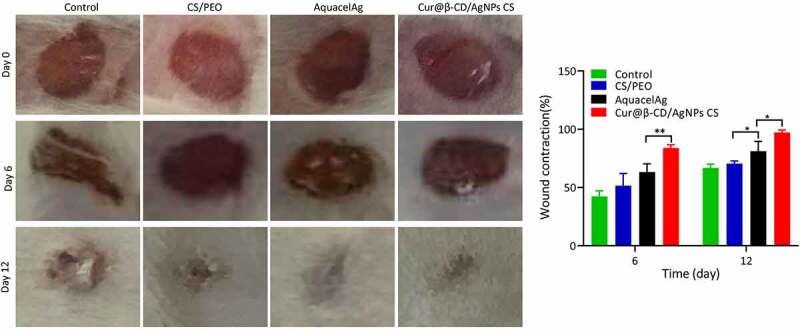
Photographic images of the extent of wound healing and graphical illustration of the changes in wound size.

The effects of nanodressing on wound healing and re-epithelialization was evaluated through comparing the wound size at different time points. As shown in Figure 6a, the size of the skin defects were remarkably minimized on day 2 in the negative group compared to groups of CS/PEO, AquacelAg and Cur@β-CD/AgNPs meshes. However, although the wound size was reduced, the reduction was caused by contract of skin due to irregular shape of wound in the blank control group without wound dress, the wounds showed triangle or irregular shapes. On the contrary, the wound shapes of CS/PEO, AquacelAg and Cur@β-CD/AgNPs groups kept round. Prominent decrease in the defect of skin was found in Cur@β-CD/AgNPs and AquacelAg nanofibrous dresses compared to the CS/PEO nanofibrous dressing on day 6 and 12. Compared to commercial AquacelAg, the wound closure rate of Cur@β-CD/AgNPs CS dressings showed enhanced wound closure rate (Figure 6). After wound healing, the skin of Cur@β-CD/AgNPs CS dressing group were more smooth with less scar, while the skin of the other groups showed irregular healing scars, with uneven skin surface. It is worth noting that Cur@β-CD/AgNPs CS dressing attached easily to the wounds to keep the wound desirably hydrated.

### 3.7. Histology analysis

**Figure 7.**
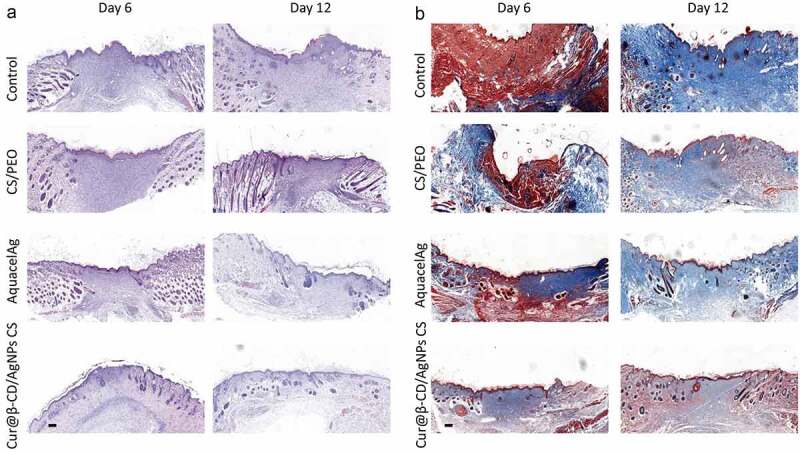
Effects of CS/Cur@β-CD/AgNPs nanofibrous on wound closure and re-epithelialization. (a, b) H&E and MT stain images were presented at day 6 and day 12; Scale bars represent 200 μm.

The histopathology of wound samples was examined by H&E stain. The histological data demonstrated that Cur@β-CD/AgNPs CS dressing as well as the AquacelAg dressing wreaked the degree necrosis in comparison with the group of CS/PEO (Figure 7a). Cur@β-CD/AgNPs CS dressing and the AquacelAg dressing induced earlier granulation tissue formation. Additionally, adhibition of Cur@β-CD/AgNPs CS dressings to the skin wound lessened the quantity of inflammatory cells in comparison with AquacelAg dressing, and the infiltrated inflammatory cells were completely attenuated at day 12 (Figure 7a). Cur@β-CD/AgNPs CS dressings displayed earlier epithelization and thicker epidermis compared to AquacelAg. The deposition of collagen was evaluated by MT stain at day 6 and 12, Cur@β-CD/AgNPs CS dressings displayed abatement of collagen deposition and uniform distribution of collagen (Figure 7b).

## 4. Conclusion

In our experiment, we prepared Cur@β-CD/AgNPs which consisted of silver in the core and curcumin on the surface with one-pot synthesis. β-CD increases the stability of nano materials and β-CD has been verified as hydrophobic cavity to load curcumin, a representative of hydrophobic phytochemicals. In our study, the release rate of curcumin in the acidic environment which was similar with bacterial infection microenvironment was significantly faster than that in physiological environment. The combination of released curcumin and exposed silver showed stronger antibacterial activity against *P. aeruginosa*, *S. aureus* and *E. coli* compared to commercial AgNPs dressing. On the base of CS and Cur@β-CD/AgNPs, we successfully established a novel effective antibacterial electrospinning wound dressing. The swelling ratio of CS/Cur@β-CD/AgNPs nanofibers was 432%, indicating that higher water absorption capability involved in maintaining a moist environment via removing excess exudates. Electrospun nanofiber as a scaffold based on CS and curcumin improved bioactivity for promoting tissue regeneration, curcumin could also promote regeneration of skin wound by regulating angiogenesis and promoting proliferation of surrounding tissue and abate scar tissue formation as confirmed be in vivo experiment. All in all, our experiment offered an efficient way for producing well-organized CS/Cur@β-CD/AgNPs nanofibrous scaffolds possessed of exceptional powers to kill bacteria and promote wound healing with less scar formation.

## Highlights

1. Cyclodextrin increases the stability of nanomaterials and loads hydrophobic curcumin.

2. Acid-responsive Cur@β-CD/AgNPs showed stronger antibacteria effects.

3. Both curcumin and degraded chitosan promote wound healing with less scar formation.
